# Identification of Upland Rice Genotypes Resistant to Neck Blast Disease: A Systematic Review of Field and Greenhouse Studies

**DOI:** 10.3390/genes17020183

**Published:** 2026-01-31

**Authors:** Ojuka Jonathan, Joao Bila, Arsenio Ndeve, Lamo Jimmy

**Affiliations:** 1 Crop Production Department, Eduardo Mondlane University, Avenida Julius Nyerere, nr. 3453, Maputo 1100, Mozambique; ojukajonathan@gmail.com (O.J.); ndevegod@gmail.com (A.N.); 2 Centre of Excellence in Agrifood Systems and Nutrition, 5o Floor, Rectory Building, Praça 25 de Junho, Maputo 1100, Mozambique; jbilay@gmail.com; 3 Department of Crop Protection, Faculty of Agronomy and Forestry Engineering, Eduardo Mondlane University, Avenida Julius Nyerere, nr. 3453, Maputo 1100, Mozambique; 4 National Crops Resources Research Institute, Namulonge, Kampala P.O. Box 7084, Uganda

**Keywords:** *Magnaporthe oryzae*, neck blast resistance, rainfed cropping systems, genotype screening, field and greenhouse evaluation, quantitative trait loci, marker-assisted selection

## Abstract

This systematic review synthesizes evidence on upland rice (*Oryza sativa* L.) genotypes resistant to neck blast disease caused by *Magnaporthe oryzae*, focusing on resistant lines, screening methods, and genetic factors underlying resistance. Empirical studies published in English between 1980 and 2025 were identified through searches of PubMed, ScienceDirect, Google Scholar, and grey literature, with final searches completed on 31 October 2025. Eligible studies evaluated upland rice under upland or rainfed conditions. The risk of bias was assessed using a customized framework adapted from the ROBINS-I tool, and findings were synthesized narratively due to substantial methodological heterogeneity. Six studies from Asia and Africa, encompassing 248 genotypes, met the inclusion criteria. Twenty genotypes—including Kahei, Barkhe 1032, Barkhe 1035, Barkhe 2014, several NERICA lines, and BC1F4 backcross derivatives—demonstrated moderate to high resistance based on the IRRI 0–9 Standard Evaluation System. Two studies reported quantitative trait loci (qBFR4-1, qBl1, and qBl2) associated with durable resistance, highlighting the potential of QTL-based breeding. Despite limitations related to small sample sizes, heterogeneous methodologies, and limited molecular characterization, particularly for neck blast-specific resistance, this review underscores the promise of marker-assisted selection. Future research should prioritize neck blast-focused QTL validation, expanded genomic screening, harmonized screening protocols, and multi-location field trials to confirm resistance durability and agronomic performance across diverse upland environments.

## 1. Introduction

Rice (*Oryza sativa* L.) sustains over half of the world’s population, with upland rice playing a critical role in rain-fed ecosystems across Southeast Asia, sub-Saharan Africa, and Latin America [1,2]. Accounting for approximately 12% of global rice production, upland cultivation occurs predominantly on marginal lands characterized by variable soil fertility, erratic rainfall, and exposure to drought and aluminum toxicity [3,4]. Productivity remains low, averaging 2–3 tons per hectare compared to 5–6 tons for lowland varieties [5,6], with blast disease caused by *Magnaporthe oryzae* emerging as a primary biotic constraint limiting yield potential.

Blast disease manifests as leaf blast, node blast, and neck (panicle) blast, with neck blast being particularly damaging due to its direct impact on reproductive structures. Infection of the panicle neck causes partial or complete grain sterility, chalky kernels, and panicle breakage, resulting in yield losses of 10–80% under conducive conditions [7,8,9,10]. The pathogen thrives in humid upland environments, where high nitrogen levels, dense planting, and temperature fluctuations between 20 and 30 °C facilitate infection [11,12]. Upland systems are inherently more vulnerable due to the absence of flooding, which suppresses pathogen survival in lowland fields [13,14,15,16]. Chemical fungicides, while effective, raise environmental concerns, increase costs, and promote pathogen resistance [17,18,19,20,21], making host resistance breeding the most sustainable strategy [11,22,23].

Blast resistance comprises qualitative resistance, governed by major R-genes conferring race-specific immunity, and quantitative resistance, involving multiple QTLs providing broad-spectrum durability [23,24]. Qualitative resistance is prone to breakdown due to rapid pathogen evolution [11,25], whereas quantitative resistance offers longevity across diverse environments, exemplified by QTLs like qBFR4-1 on chromosome 4 in cultivar Kahei [23,26]. Over 122 resistance genes have been identified, with at least 39 cloned [4,21,27,28], many encoding nucleotide-binding site-leucine-rich repeat (NBS-LRR) proteins [23,29].

Screening for neck blast resistance employs field evaluations in blast-prone hotspots and greenhouse assays using controlled inoculation, typically scored on the IRRI 0–9 scale [9,10,23]. Correlations between leaf and neck blast resistance vary considerably, with some studies reporting positive associations and others documenting independent reactions [6,9,30,31,32], necessitating panicle-specific evaluations. Advanced techniques, including marker-assisted selection and doubled haploid production, accelerate resistant line development [3,4,33,34].

Despite these advances, inconsistencies in reported resistant genotypes, screening protocols, and genetic mechanisms hinder progress. Most blast resistance reviews have emphasized leaf blast or adopted gene-centric approaches prioritizing molecular cataloguing over field durability [21,27]. However, neck blast merits separate consideration for three reasons. First, its genetic architecture is partially distinct from leaf blast, with documented instances where vegetative-stage resistance does not predict panicle-stage protection [9,30,32]. Second, neck blast directly reduces grain yield through panicle sterility and breakage, making it economically more consequential than leaf blast in subsistence systems [7,8]. Third, upland rice faces unique epidemiological pressures—prolonged leaf wetness and absence of flood-mediated pathogen suppression—that limit the reliability of extrapolations from lowland systems [15,16].

This systematic review addresses the critical gap in synthesized evidence by integrating field durability assessments, upland-specific screening methodologies, and resistance consistency across environments. Applying rigorous PRISMA 2020 methodology [35,36,37,38,39], this review systematically compiles, appraises, and synthesizes empirical evidence from controlled and natural upland conditions, explicitly distinguishing neck blast from leaf blast phenotypes. The objectives are to: (i) identify upland rice genotypes with empirically validated neck blast resistance under field and greenhouse conditions, (ii) characterize screening methodologies employed for panicle-level phenotyping, and (iii) elucidate genetic factors, including QTLs and inheritance patterns, underlying observed resistance variation.

## 2. Materials and Methods

### 2.1. Eligibility Criteria

This systematic review followed a predefined protocol to ensure transparency and reproducibility in the selection of studies. Eligibility criteria were established based on the Population, Intervention/Exposure, Comparator, Outcome, and Study Design (PICOS) framework, tailored to the review’s focus on identifying upland rice genotypes resistant to neck blast disease through field and greenhouse evaluations [35].

Studies were included if they focused on upland rice (*Oryza sativa* L.) genotypes, such as traditional varieties, improved lines, breeding populations, or wild relatives evaluated specifically in upland or rain-fed conditions. Genotypes from other rice ecotypes, like lowland or irrigated varieties, were considered only if they were explicitly tested under upland-like simulations. Evaluations had to assess resistance to neck blast (panicle blast) caused by *Magnaporthe oryzae*, encompassing natural infections, artificial inoculations, or molecular characterizations linked to neck blast phenotypes. Studies with or without comparators were eligible, though preference was given to those incorporating susceptible checks, resistant standards (for example, varieties with known R-genes like *Pi9* or *Pi2*), or multi-environment trials for benchmarking resistance levels.

Primary outcomes required the identification of resistant genotypes, rated as resistant or highly resistant on standard scales like the 0–9 SES, along with yield loss estimates due to neck blast, genetic markers or QTLs associated with resistance, and details on screening methodologies. Secondary outcomes included correlations between leaf and neck blast resistance, pathogen isolate variability, and environmental influences on resistance expression. Eligible study designs comprised empirical research reporting original data from field trials, such as hotspot screenings or multi-location tests, or greenhouse experiments involving controlled inoculations or seedling trays. Peer-reviewed journal articles, conference proceedings, theses, and technical reports published in English from January 1980 to September 2025 were considered to capture both historical and recent advancements. Additionally, studies needed to provide sufficient detail on methodology, including inoculation techniques and rating scales, as well as results like genotype lists and resistance scores, to enable data extraction and synthesis [36].

Studies were excluded if they concentrated exclusively on leaf blast, node blast, or other rice diseases without specific data on neck blast. Evaluations limited to non-upland rice systems, such as purely lowland or flooded conditions, were omitted unless upland adaptations were simulated and reported. Non-empirical works, including narrative reviews, opinion pieces, or modeling studies without experimental validation, did not qualify. Research lacking quantitative or qualitative resistance data, such as mere disease incidence reports without genotype-specific assessments, was also excluded. Publications in languages other than English or those with inaccessible full texts after reasonable efforts, like interlibrary loans or author contacts, were not included. Duplicate reports of the same dataset were handled by retaining the most comprehensive or recent version. For syntheses, studies were grouped to facilitate narrative and thematic analyses, as meta-analysis was not feasible due to heterogeneity in screening protocols and reporting standards [37].

The primary grouping distinguished between field-based studies, which emphasized natural inoculum and multi-seasonal durability, and greenhouse-based studies, which focused on controlled conditions for precise phenotyping. Within these categories, subgroups were formed based on geographical regions, such as Asia, Africa, and Latin America, to account for agroecological variations and pathogen diversity. Additional groupings addressed resistance type, differentiating qualitative or major gene resistance from quantitative or QTL-based resistance; screening intensity, such as single versus multi-isolate challenges; and genetic focus, including studies reporting cloned genes or applications of marker-assisted selection. This stratification allowed for the identification of consistent resistant genotypes across contexts, highlighting methodological trends and exposing knowledge gaps for future research [38].

### 2.2. Information Sources

The search for relevant studies was conducted across multiple electronic databases, and grey literature sources to ensure comprehensive coverage of the literature on upland rice resistance to neck blast, adhering to recommended practices for systematic reviews in agricultural sciences [39]. Key databases included PubMed for biomedical and pathology-related studies, ScienceDirect for peer-reviewed journal articles in agronomy and crop science, and Google Scholar for broad academic coverage including theses and conference papers. Additionally, reference lists from included studies and seminal review articles on rice blast resistance were hand-searched for supplementary citations. Grey literature, including theses from ProQuest Dissertations and Theses Global and reports from national agricultural research institutes, was screened to minimize publication bias. The search dates were from January 1980 to September 2025 and searches were confined to English language publications. The final searches across all sources were completed on 31 October 2025. A total of 420 records were initially identified from the primary databases: 200 from PubMed, 10 from ScienceDirect, and 210 from Google Scholar. Subsequent deduplication using Rayyan software [40] removed 7 redundant records before full-text screening commenced.

### 2.3. Search Strategy

A comprehensive and reproducible search strategy was developed to identify empirical field and greenhouse studies that evaluated upland *Oryza sativa* genotypes for resistance to neck (panicle) blast caused by *Magnaporthe oryzae*. The strategy was constructed a priori using the PICOS framework to define the population, exposure, outcomes, and eligible study designs and was iteratively refined through pilot searches to balance sensitivity and specificity [35]. Search planning and reporting followed the PRISMA guidance for systematic reviews to ensure transparency and replicability [39]. Search terms combined controlled vocabulary and free-text terms covering the crop (*Oryza sativa*, rice), pathogen (*Magnaporthe oryzae*, *Pyricularia oryzae*), target organ (neck, panicle), ecotype (upland, rain-fed), screening environment descriptors (field, hotspot, greenhouse, glasshouse, controlled inoculation), and molecular/genetic keywords (SNP, single nucleotide polymorphism, QTL, marker, R-gene).

Electronic searching was conducted across multiple bibliographic databases and platforms relevant to agricultural science and plant pathology, including PubMed for biomedical/pathology literature and ScienceDirect for agronomy and crop science journals. Google Scholar was used to extend coverage to theses, conference papers, and other grey literature sources, and ProQuest Dissertations and Theses Global and national agricultural research institute repositories were searched to capture dissertations and technical reports. The search strings were adapted to PubMed database’s syntax and indexing. For example, the search string was: (“upland rice” OR OR “rainfed rice“ OR “Oryza sativa") AND (“Magnaporthe oryzae“ OR “neck blast“ OR “panicle blast“ OR “Pyricularia oryzae“) AND (“plant resistance“ OR resistance OR “resistant genotype*“ OR QTL OR “R gene“) AND (“field trial*“ OR greenhouse OR screening) NOT (“lowland rice“ OR irrigated). On the other hand, title/abstract/keyword fields were used in ScienceDirect and Google Scholar.

### 2.4. Selection Process

Study selection proceeded through a two-stage screening process (title/abstract followed by full-text assessment) implemented to ensure consistent and reproducible application of the pre-specified eligibility criteria (See Section 2.1). Following export from the database interfaces into reference management software and initial deduplication, records were uploaded to Rayyan for blinded screening and collaborative management [40]. Deduplication removed 7 redundant records from the 420 records retrieved during the primary electronic searches; the remaining unique records entered the title/abstract screening stage.

Title and abstract screening was performed independently by two reviewers. Prior to formal screening, the review team conducted a calibration exercise using a random sample of records to verify shared understanding of the inclusion/exclusion criteria, to refine the screening form, and to ensure consistency in judgement. During the main screening phase each reviewer classified records as “include”, “exclude”, or “uncertain”; Rayyan’s interface was used to blind reviewers to one another’s decisions and to facilitate the management of conflicts [40]. Records judged as “include” or “uncertain” by either reviewer progressed to full-text assessment. Rayyan was employed for deduplication, blinded dual screening, and conflict tracking.

### 2.5. Data Collection Process

Data extraction from the included studies was conducted using a standardized electronic form developed a priori and piloted on a subset of eligible reports to ensure clarity, completeness, and consistency in capturing relevant information. The form was designed in Microsoft Excel to facilitate structured data entry and was tailored to the review’s objectives, encompassing key domains such as study characteristics, screening methodologies, genotype details, resistance outcomes, and genetic factors (see Section 2.6 for specific data items). Two reviewers independently extracted data from each included report to minimize bias and enhance accuracy. Prior to the main extraction phase, a calibration exercise was performed on five randomly selected studies to align interpretations and resolve any ambiguities in the form’s application.

For each report, reviewers worked separately, documenting extractions in duplicate forms before comparing results. Discrepancies between the extracted data were identified through comparison and resolved via discussion between the two reviewers; if consensus could not be reached, a third senior reviewer was consulted for arbitration. No automation tools were employed for the data extraction process itself, as it relied on manual interpretation of textual and tabular content to ensure specific understanding of complex agronomic and genetic details. However, Rayyan software, previously used for screening [40], was leveraged for organizing full-text PDFs and tracking extraction progress, facilitating collaborative workflow management among the review team. All extracted data were compiled into a central database for subsequent synthesis and analysis.

### 2.6. Data Items

Data items were predefined based on the review’s objectives and structured to capture both outcome-related and contextual information from eligible studies. Extraction focused on quantitative and qualitative elements essential for synthesizing evidence on upland rice resistance to neck blast, with all items documented in a piloted extraction form.

For primary outcomes, data were sought on the identification of resistant upland rice genotypes, defined as those classified as resistant (R), moderately resistant (MR), or highly resistant (HR) based on standard rating scales such as the International Rice Research Institute’s Standard Evaluation System (SES) 0–9 scale (where scores of 0–3 indicate resistance) or equivalent visual symptom-based metrics (e.g., percentage of infected panicles or disease severity index) [41]. This included any reported resistant genotypes, along with associated yield loss estimates attributable to neck blast (e.g., percentage reduction in grain yield or filled grains per panicle under infected conditions). Additional primary outcomes encompassed genetic markers or QTLs linked to neck blast resistance (e.g., specific R-genes like *Pi9* or QTLs like *qBFR4-1*, including chromosomal locations and effect sizes where available), and details on screening methodologies (e.g., inoculation techniques, such as spore suspension concentration in greenhouse assays or natural hotspot exposure in field trials, along with evaluation timings like days after heading).

Secondary outcomes included correlations between leaf and neck blast resistance (e.g., Pearson’s correlation coefficients or qualitative associations), pathogen isolate variability (e.g., race or avirulence gene profiles of *Magnaporthe oryzae* strains used), and environmental influences on resistance expression (e.g., impacts of temperature, humidity, or nitrogen levels on disease severity). All results compatible with these outcome domains were sought from each study, including multiple measures (e.g., different rating scales), time points (e.g., initial vs. final disease assessments), and analyses (e.g., single-season vs. multi-year data or isolate-specific vs. mixed-inoculum results). No selective collection methods were applied; if multiple results were reported for an outcome (e.g., resistance scores across different years or locations), all were extracted to enable comprehensive synthesis and assessment of consistency or variability.

Other variables for which data were sought included study characteristics such as publication year, geographical location (e.g., country or region like Southeast Asia or sub-Saharan Africa), study design (e.g., field trial, greenhouse experiment, or combined), number of genotypes evaluated, comparator details (e.g., susceptible checks like IR64 or resistant standards like Moroberekan), funding sources (e.g., institutional, governmental, or private grants), and author affiliations. Genotype-specific variables encompassed origin (e.g., traditional landrace, improved cultivar, or breeding line), and agronomic traits (e.g., maturity duration or yield potential under non-diseased conditions). Methodological variables included pathogen inoculum details (e.g., isolate source, concentration, or application method), environmental conditions (e.g., temperature ranges or rainfall patterns in field studies), and statistical analyses (e.g., ANOVA for resistance differences or heritability estimates). For missing or unclear information, assumptions were made conservatively: for instance, if resistance ratings were not explicitly linked to a scale, it was assumed to align with the SES 0–9 system if contextually implied (e.g., based on cited IRRI protocols). All assumptions were documented in the extraction form and considered during risk of bias assessment to mitigate potential misinterpretation.

### 2.7. Study Risk of Bias Assessment

The risk of bias (RoB) in included studies was assessed to evaluate the internal validity of the evidence and inform the strength of synthesized findings. Given the predominance of experimental (field and greenhouse) rather than randomized controlled trial designs in agronomic research, a customized RoB tool was developed a priori, adapted from the Risk of Bias in Non-randomized Studies of Interventions (ROBINS-I) tool [42], tailored to the context of plant pathology and crop breeding studies. ROBINS-I was selected over alternative frameworks, such as the Cochrane Risk of Bias 2.0 tool, which presumes randomization at the participant level, or the SYRCLE tool for animal studies because it explicitly accommodates observational and quasi-experimental designs common in field trials where genotype allocation often follows augmented or incomplete block designs rather than strict randomization [42]. Additionally, ROBINS-I’s domain structure (covering confounding, selection, intervention deviations, missing data, measurement, and reporting) aligns closely with validity threats in disease resistance evaluations, such as environmental heterogeneity and non-blinded phenotypic scoring, whereas agronomy-specific tools like those for variety trial meta-analysis prioritize genotype × environment interactions over internal validity domains [37,38].

The customized tool comprised seven domains relevant to field and greenhouse evaluations of disease resistance: (1) selection of genotypes (e.g., representativeness and randomization in trial design), (2) exposure to pathogen (e.g., standardization and reproducibility of inoculation methods), (3) confounding factors (e.g., control for environmental variables like temperature or humidity), (4) detection/outcome assessment (e.g., blinding of raters and reliability of scoring scales), (5) incomplete outcome data (e.g., attrition of genotypes or missing yield assessments), (6) selective reporting (e.g., completeness of resistance data across all tested lines), and (7) other biases (e.g., funding conflicts or methodological deviations). Each domain was rated as low risk, moderate risk, high risk, or unclear risk, with supporting justifications extracted from the study reports. An overall RoB judgment was assigned per study as low (all domains low), moderate (mostly low with some moderate), high (one or more high domains), or unclear (insufficient information).

To minimize subjectivity, two reviewers independently assessed RoB for each study using a standardized Microsoft Excel form piloted on three studies to refine domain definitions and scoring criteria, ensuring consistent interpretation of terms such as “adequate randomization” (defined as documented use of random number generation or systematic blocking) versus “unclear randomization” (allocation sequence not described). Assessments were conducted blindly, with discrepancies resolved through structured discussion referencing predefined resolution criteria: for example, studies lacking explicit randomization statements but employing replicated designs (e.g., RCBD) were rated “some concerns” rather than “high risk” for Domain 1, reflecting partial mitigation of selection bias. Where consensus could not be reached, a third senior reviewer arbitrated based on these criteria. Operational definitions for key domains were established: Domain 2 (pathogen exposure) required explicit reporting of inoculum concentration (e.g., spores/mL), application timing, and environmental conditions post-inoculation to achieve “low risk”; Domain 4 (outcome assessment) necessitated either documented blinding of disease scorers or use of objective metrics (e.g., digital image analysis) to avoid “high risk” for measurement bias. These procedures enhanced reproducibility and reduced reviewer-dependent variability, though residual subjectivity persisted in borderline cases—particularly for Domain 3 (confounding), where judgments about adequacy of environmental controls in field studies required qualitative interpretation of meteorological descriptions.

Important limitations of the adapted tool include: (i) absence of formal validation against a gold standard of methodological quality in rice breeding trials, as ROBINS-I was originally developed for clinical intervention studies; (ii) potential misclassification in Domain 7 (other biases), where unreported funding sources were conservatively rated “unclear” rather than assuming low risk, potentially inflating uncertainty; and (iii) limited sensitivity to design-specific biases such as spatial heterogeneity in augmented designs used by [3], which are not captured in ROBINS-I’s standard domains. Despite these constraints, the tool provided a structured, transparent framework for evaluating internal validity, with domain-level ratings enabling nuanced interpretation of evidence quality and informing subsequent certainty assessments (Section 2.11) [42].

### 2.8. Effect Measures

Effect measures were predefined a priori to structure the presentation of resistance outcomes and facilitate transparent interpretation of neck blast resistance patterns across field and greenhouse evaluations, adhering to PRISMA 2020 guidelines for systematic reviews [39] and recommendations from the Cochrane Handbook for Systematic Reviews of Interventions [43]. Given the nature of disease resistance outcomes reported in rice blast studies, effect measures were categorized according to outcome type: binary classifications (resistant versus susceptible) and continuous quantitative assessments (mean severity scores or disease indices). For binary outcomes, such as the classification of genotypes as resistant (R), moderately resistant (MR), or susceptible (S) based on categorical thresholds derived from the International Rice Research Institute (IRRI) Standard Evaluation System (SES) 0–9 scale (where scores 0–3 indicate resistance, 4–6 moderate resistance, and 7–9 susceptibility) [41], the Risk Ratio (RR) was identified as the preferred effect measure when incidence data were available, enabling calculation of the relative likelihood of resistance between genotypes or screening environments. The Risk Difference (RD) was considered an alternative for expressing absolute differences in resistance proportions. However, extraction from included studies revealed that only ref. [9] reported neck blast outcomes in an incidence-based format (resistant: 0–15%, moderately resistant: 15.1–30%, moderately susceptible: 30.1–50%, susceptible: 50.1–100%), but exact numerical values and variance estimates were not provided, precluding formal RR or RD calculation. Consequently, binary outcomes were synthesized descriptively, tabulating the number and percentage of resistant genotypes identified per study.

For continuous outcomes, primarily mean neck blast severity scores measured on the IRRI 0–9 scale, Mean Difference (MD) was selected as the effect measure when studies employed a common metric [41]. MD, calculated as the difference in mean severity scores between resistant genotypes and susceptible checks or between experimental conditions, provided an interpretable measure of resistance magnitude in score units. Ref. [17] reported panicle severity scores on the IRRI scale for 13 upland genotypes, with most scoring 0.00 compared to susceptible checks NIMBAM 10 (7.50) and NIMBAM 11 (6.66), yielding theoretical MDs of approximately 7.50 and 6.66 scale units, respectively; however, standard deviations or standard errors were not reported, preventing calculation of confidence intervals or statistical significance. Where variance data were incomplete, as observed across all included studies [3,4,9,10,17,23], MDs were presented descriptively without precision estimates. The Standardized Mean Difference (SMD) was considered for outcomes measured on different severity scales or percentage-based indices; however, its application was precluded by the absence of both variance parameters and heterogeneity in severity metrics across studies.

Notably, formal meta-analysis incorporating pooled effect estimates was not feasible for the majority of studies due to: (i) missing standard deviations or standard errors for severity scores, which prevented calculation of weighted MD or SMD; (ii) absence of neck blast-specific outcomes in four studies [3,4,10,23], which reported only leaf blast severity or QTL mapping data without panicle-level disaggregation; and (iii) methodological diversity in inoculation protocols, pathogen isolates, and environmental conditions, which introduced clinical and statistical heterogeneity unsuitable for pooling [37]. Consequently, effect measures served primarily to structure descriptive synthesis, stratified by study setting (field trials [17] versus greenhouse inoculations [9]) and outcome type (binary classifications versus continuous scores), ensuring transparent reporting of resistance patterns while acknowledging limitations imposed by incomplete primary data reporting, consistent with best practices for systematic reviews in plant pathology [37,38].

### 2.9. Synthesis Methods

#### 2.9.1. Eligibility for Synthesis

Studies were deemed eligible for synthesis if they reported empirical data on neck blast resistance in upland rice genotypes, assessed through field trials or greenhouse inoculations, with sufficient methodological detail to enable extraction of resistance outcomes. Following the pre-specified PICOS framework (Section 2.1), eligibility for each synthesis group was determined by tabulating (see Table A3) study characteristics, including genotype populations, screening environments (field versus greenhouse), outcome types (binary classifications versus continuous severity scores), and availability of neck blast-specific data, and comparing these against the planned synthesis categories [39]. Of the six included studies, only two reported neck blast outcomes suitable for structured synthesis: ref. [17] provided continuous panicle severity scores from field evaluations, while ref. [9] reported incidence-based classifications from greenhouse screenings. The remaining four studies [3,4,10,23] were excluded from quantitative synthesis due to absence of neck blast-specific assessments, limiting their contribution to leaf blast evaluations, QTL mapping without phenotypic disaggregation, or genetic diversity analyses focused on vegetative stages. This determination ensured that synthesis groups aligned with the review’s primary objective of identifying neck blast resistance while maintaining transparency regarding data limitations [43].

#### 2.9.2. Data Preparation

Data preparation required several adaptations to address missing summary statistics and heterogeneous reporting formats. For continuous outcomes, where ref. [17] reported mean panicle severity scores on the IRRI 0–9 scale without standard deviations or standard errors, effect estimates (mean differences) were calculated descriptively by subtracting genotype scores from susceptible check means, but confidence intervals could not be derived. For binary outcomes, ref. [9] presented neck blast incidence data graphically across four categorical resistance thresholds (resistant: 0–15%, moderately resistant: 15.1–30%, moderately susceptible: 30.1–50%, susceptible: 50.1–100%), necessitating visual estimation of approximate incidence percentages from bar charts due to the absence of tabulated numerical values. These estimated values were recorded with explicit notation of their approximate nature in the extraction database. No formal data conversions (e.g., between severity indices or scales) were required, as both studies employed IRRI-compatible metrics [43]. Where genotype sample sizes were unclear, conservative assumptions were made based on experimental designs: for ref. [17], approximately 75 plants per genotype (25 plants × 3 replications) were inferred from the randomized complete block design description; for ref. [9], 4 seedlings per genotype were explicitly stated. All assumptions and approximations were documented in the extraction form and considered during risk of bias assessment to maintain transparency [40].

#### 2.9.3. Tabulation and Visualization

**Figure 1:**
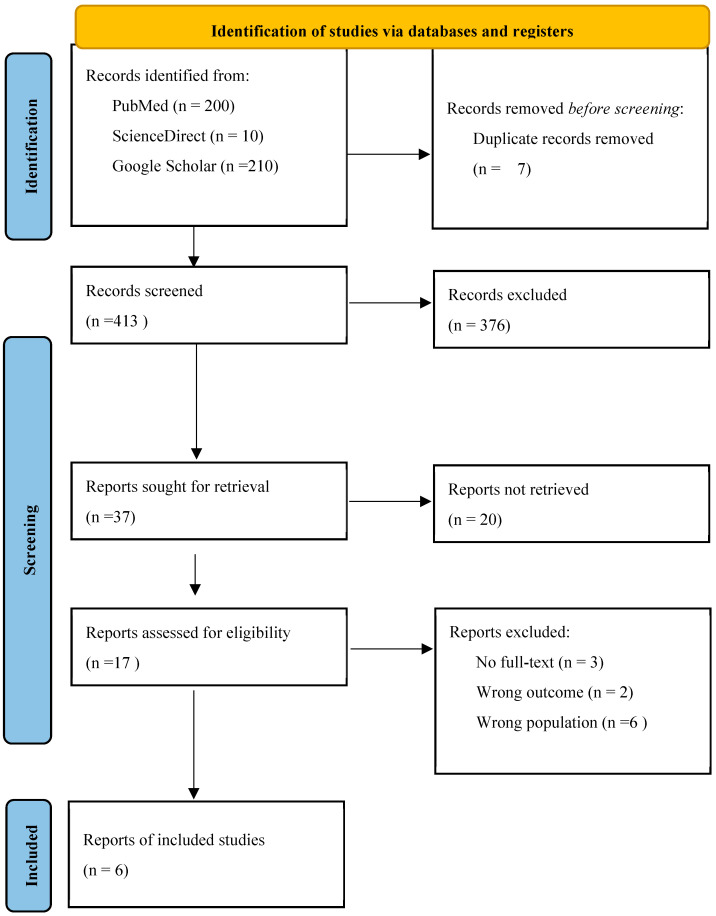
PRISMA flow diagram showing the identification of upland rice genotypes resistant to neck blast disease. Source: [38].

Results of individual studies were tabulated in structured summary tables (Table A1 and Table A2) organized by study characteristics, genotype information, and extracted outcome data, facilitating systematic comparison across screening environments and resistance metrics. Table 1 presented study-level metadata (author, year, country, setting, design, replications) alongside genotypes evaluated and comparators employed. Table A1 displayed risk of bias assessments across predefined domains (D1–D5) with overall judgments for each study, enabling visual identification of methodological strengths and limitations. For neck blast outcomes specifically, a dedicated Table A2 was constructed to display mean severity scores or incidence categories for resistant genotypes and susceptible checks from [17] and [9], respectively, alongside sample sizes and scoring scales. The geographical distribution of study sites was visualized using a world map (Figure 1 to illustrate regional representation and identify evidence gaps (e.g., absence of Latin American studies). The PRISMA flow diagram (Figure 1) depicted the study selection process from initial database searches (*n* = 420 records) through deduplication, screening, and final inclusion (n = 6 studies), with reasons for exclusion documented at the full-text assessment stage [38]. These visualization methods enhanced transparency and interpretability of the evidence base.

#### 2.9.4. Methods for Synthesis

Given the paucity of neck blast-specific data (*n* = 2 studies) and absence of variance estimates necessary for meta-analysis, a narrative synthesis approach was adopted as the primary method for integrating findings, consistent with guidance for reviews where statistical pooling is precluded by heterogeneity or incomplete reporting [37,43]. Narrative synthesis proceeded thematically, organizing evidence into predefined categories addressing the review’s research questions: (i) identification of resistant genotypes (stratified by field versus greenhouse screening), (ii) screening methodologies employed (including inoculation protocols, disease scoring systems, and evaluation timings), and (iii) genetic factors influencing resistance variation (distinguished by molecular versus phenotypic characterization). Within each theme, results were synthesized descriptively by extracting common patterns (e.g., recurrent identification of Barkhe series genotypes across [9,10]), noting divergent findings (e.g., variable correlations between leaf and neck blast resistance), and contextualizing outcomes relative to risk of bias assessments. For the two studies reporting neck blast data, effect measures (mean differences for ref. [17]; resistance classifications for ref. [9]) were presented alongside qualitative interpretations of resistance magnitudes relative to susceptible checks and IRRI scale thresholds. No formal meta-analysis models (e.g., random-effects or fixed-effects pooling) were applied, nor were statistical heterogeneity metrics (I^2^, τ^2^) calculated, as insufficient studies with comparable outcomes and variance data existed to justify such analyses [43]. The rationale for narrative synthesis centered on preserving the context specific of each study’s findings while transparently acknowledging data limitations that prevented quantitative pooling [38].

#### 2.9.5. Investigations of Heterogeneity

Although formal statistical assessment of heterogeneity was not feasible due to the absence of meta-analysis, potential sources of variation among study results were explored qualitatively through stratified synthesis and comparative analysis of study characteristics [43]. Subgroup analyses were conducted by screening environment (field-based evaluations [17] versus greenhouse inoculations [9]) to assess whether resistance expression differed systematically between natural and controlled conditions, with findings suggesting lower severity scores under greenhouse settings potentially attributable to standardized humidity and temperature regimes. Geographical subgroups (Asian studies: n = 5; African studies: n = 1) were compared to identify regional patterns in pathogen virulence or genotype performance, revealing consistent resistance in Barkhe genotypes across Nepal [9] and Malaysia [10] but limited African data for robust comparison. Methodological heterogeneity was evaluated by tabulating inoculation methods (e.g., spore suspension concentrations ranging from 10^5^ spores/mL to unspecified field spray applications), disease scoring systems (IRRI 0–9 scale in [17] versus incidence thresholds in [9]), and pathogen isolate diversity (single versus multiple races), with narratives highlighting how these variations likely contributed to differential resistance outcomes. Risk of bias patterns (e.g., “some concerns” in field studies [17] versus “low risk” in greenhouse studies [9]) were also examined as potential explanatory factors. No meta-regression was performed due to insufficient data points for modeling relationships between study-level covariates and effect sizes.

#### 2.9.6. Sensitivity Analyses

Formal sensitivity analyses, such as exclusion of studies with high risk of bias or alternative effect measure calculations, were not conducted because the limited number of studies reporting neck blast outcomes (n = 2) precluded the meaningful assessment of result robustness through iterative exclusion or model adjustments [43]. However, the review incorporated transparency measures analogous to sensitivity analysis principles: results were presented with and without studies exhibiting “some concerns” in risk of bias assessments [17], and narrative synthesis explicitly noted where conclusions relied on single-study evidence or approximated data (e.g., visual estimation of incidence percentages from [9]). For studies excluded from neck blast synthesis due to outcome mismatch [3,4,10,23], their contributions to broader resistance characterization (e.g., genetic markers, breeding methodologies) were synthesized separately to ensure comprehensive evidence integration without conflating distinct outcome domains. This approach maintained interpretive caution regarding the strength and generalizability of synthesized findings, acknowledging that conclusions about neck blast resistance were anchored to a narrow evidence base vulnerable to individual study limitations [37,38].

### 2.10. Reporting Bias Assessment

The risk of bias due to missing results (reporting biases) was assessed to evaluate whether the synthesized evidence might be distorted by selective non-publication of studies or selective non-reporting of outcomes within studies, following PRISMA 2020 guidance [39] and recommendations from the Cochrane Handbook [43]. Given the small number of included studies (n = 6) and the even smaller subset reporting neck blast outcomes (n = 2), formal statistical assessment of publication bias through funnel plot asymmetry or Egger’s test was not feasible, as such methods require a minimum of 10 studies for reliable interpretation [43]. Instead, reporting bias was evaluated qualitatively through multiple complementary approaches.

For instance, the comprehensiveness of the search strategy was assessed to gauge the likelihood of missing relevant studies. Searches spanned multiple databases (PubMed, ScienceDirect, Google Scholar) and grey literature sources (ProQuest Dissertations and Theses Global, institutional repositories), with no language restrictions beyond English, and were supplemented by hand-searching reference lists of included studies and seminal reviews. Despite these efforts, 12 studies identified at full-text screening were excluded due to inaccessibility [42,43,44], raising moderate concern that unpublished or difficult-to-access research—particularly from non-English-speaking regions or national agricultural institutes with limited digital dissemination—may have been missed. The predominance of Asian studies (n = 5) versus African (n = 1) and complete absence of Latin American research, despite upland rice’s regional importance [1], suggests potential geographical reporting bias, possibly reflecting underfunding of blast research in certain regions or preference for publishing in non-indexed local journals.

Similarly, selective outcome reporting within studies was examined by comparing extracted outcomes against stated objectives in Section 2. Notably, four of six included studies [3,4,10,23] did not report neck blast outcomes despite investigating blast resistance broadly, instead focusing exclusively on leaf blast or QTL mapping without panicle-level phenotyping. This pattern raises concern about selective outcome reporting bias, wherein negative or non-significant neck blast results may have been obtained but not reported, potentially because neck blast proved more difficult to induce consistently in greenhouse settings or because it showed weaker genetic associations than leaf blast. For instance, ref. [23] described a “novel evaluation system” for field resistance but presented only leaf blast lesion scores without explaining whether panicle assessments were attempted, suggesting possible post-hoc outcome selection. Similarly, refs. [3,4] evaluated blast resistance at multiple growth stages but reported only seedling-stage leaf blast severity, with no justification for omitting reproductive-stage assessments. The absence of study protocols or trial registrations for all included studies precluded formal comparison of planned versus reported outcomes, amplifying uncertainty about selective reporting [43].

### 2.11. Certainty Assessment

The certainty (or confidence) in the body of evidence for each outcome was assessed using an adapted framework informed by the Grading of Recommendations Assessment, Development and Evaluation (GRADE) approach [44], tailored to the context of observational agronomic studies rather than clinical interventions, as recommended for systematic reviews in plant pathology [37,38]. Certainty was evaluated separately for the review’s three primary outcomes: (i) identification of resistant genotypes, (ii) screening methodologies, and (iii) genetic factors influencing resistance, with ratings assigned as high, moderate, low, or very low based on five domains: risk of bias, inconsistency, indirectness, imprecision, and publication bias [43,44].

Outcome 1: Identification of Upland Rice Genotypes Resistant to Neck Blast Disease

Certainty in the evidence identifying resistant genotypes was rated as low. The starting rating was moderate (observational studies inherently carry limitations relative to randomized trials), which was then downgraded by two levels due to serious concerns across multiple domains. The risk of bias was rated as serious: while two studies reporting neck blast outcomes exhibited overall low risk [9] or some concerns [17], methodological limitations included lack of blinding in disease assessments, incomplete randomization in field trials, and absence of variance reporting that precluded precision estimates for resistance magnitudes.

Inconsistency was not formally assessable due to limited overlap in the genotypes evaluated (only Barkhe series appeared across multiple studies [9,10], showing consistent resistance), but the divergent outcome metrics (continuous severity scores in ref. [17] versus categorical incidence in ref. [9]) precluded direct comparisons, introducing uncertainty about whether genotypes would perform consistently under different evaluation systems.

Indirectness was moderate: both studies evaluated upland rice under relevant conditions (field trials in Kenya [17]; greenhouse inoculations mimicking field pressures in Nepal [9]), but generalizability to other agroecologies (e.g., acidic soils in Latin America, subtropical climates in Southeast Asia) remained uncertain due to narrow geographical representation and potential genotype × environment interactions not captured in single-season or single-location assessments.

Imprecision was very serious: the absence of standard deviations, confidence intervals, or sample size justifications meant that resistance estimates lacked quantifiable precision, and small sample sizes (e.g., 4 seedlings per genotype in [9]; unspecified replication in [17]) raised concerns about effect stability, particularly for genotypes with intermediate resistance (e.g., Niwah with severity score 3.33 in [17]).

Outcome 2: Screening Methodologies for Assessing Neck Blast Resistance

Certainty in the evidence characterizing screening methods was rated as moderate. The starting rating was moderate, downgraded by one level for serious imprecision but not further reduced due to relatively low concerns in other domains. The risk of bias was acceptable: methodological descriptions in refs. [9,17] provided sufficient detail on inoculation protocols (spore concentrations, application timings, environmental conditions) and disease scoring systems (IRRI 0–9 scale [41]; incidence thresholds), enabling replication, and both studies employed replicated designs (3 replications in ref. [17]; 2-year evaluation in ref. [9]) that enhanced the reliability of procedural assessments.

Inconsistency was low: both field spray inoculation [17] and greenhouse spore suspension application [9] successfully induced neck blast, demonstrating feasibility across settings, and the use of IRRI-compatible metrics facilitated comparability despite different outcome formats. Indirectness was minimal: the evaluated methods directly addressed the review question regarding protocols applicable to upland rice neck blast screening, with clear relevance to breeding program implementations.

Imprecision was serious: while methods were described qualitatively, quantitative validation of method reliability (e.g., inter-rater agreement for disease scoring, correlation coefficients between greenhouse and field results within the same study) was not reported, limiting conclusions about measurement precision; ref. [9] noted a weak positive correlation (r ≈ 0.30, estimated from text) between leaf and neck blast, but confidence intervals were absent, precluding the robust assessment of method concordance.

Outcome 3: Genetic Factors Influencing Neck Blast Resistance Variation

Certainty in the evidence on genetic factors was rated as very low. The starting rating was moderate, downgraded by three levels due to very serious concerns in multiple domains. The risk of bias was moderate: the two studies providing molecular data [4,23] exhibited low overall risk in their respective designs (marker-assisted selection with rigorous genotyping in ref. [4]; QTL fine-mapping with validated markers in ref. [23]), but neither reported neck blast phenotypes, limiting their direct contribution to genetic explanations of panicle resistance specifically—only leaf blast QTLs (qBFR4-1, qBl1, qBl2) were characterized, with neck blast associations inferred indirectly through breeding outcomes or literature references.

Inconsistency was not assessable due to the absence of overlapping genetic loci or populations between the two molecular studies, and the four studies lacking molecular data [3,9,10,17] provided only phenotypic or inheritance-based inferences (e.g., F2 segregation ratios in ref. [17]), which could not be triangulated against molecular findings to assess concordance. Indirectness was very serious: the reported QTLs (qBFR4-1 on chromosome 4 [23]; qBl1 and qBl2 on chromosomes 1 and 2 [4]) were characterized exclusively for leaf blast resistance in greenhouse assays, with extrapolation to neck blast resistance based on assumptions of shared genetic architecture rather than direct phenotypic validation; this introduces substantial uncertainty, as leaf and neck blast can be controlled by distinct loci or exhibit genotype-specific decoupling, as noted in ref. [9]’s partial correlation findings.

Imprecision was very serious: even for the characterized leaf blast QTLs, effect sizes (e.g., R^2^ = 25% for qBFR4-1 [23]) lacked confidence intervals or validation across independent populations for neck blast specifically, and the absence of genome-wide association studies or multi-QTL validation meant that the contribution of uncharacterized loci to observed resistance variation remained unknown, potentially large, and unquantified.

## 3. Results

### 3.1. Study Selection

Searches and study selection were performed following PRISMA guidance [39]. A PRISMA flow diagram summarizing identification, screening, eligibility assessment, and final inclusion is presented in Figure 1. From the studies and extraction table provided for this review, six primary studies met the review’s inclusion criteria and were retained for synthesis. The six included studies [3,4,9,10,17,23] originate from six countries across Asia and Africa (Indonesia, Kenya, Japan, Thailand, Nepal, Malaysia) and collectively evaluated a broad set of upland rice genotypes for resistance to neck (panicle) blast. Study designs and methods varied: four studies used greenhouse-based inoculation systems [3,4,9,23], one study applied field screening exclusively [17], and one combined field and greenhouse approaches [10]. Molecular approaches ranged from QTL mapping and graphical genotyping [23] to marker-assisted backcross selection using known QTL/genes [4]. Several studies did not report the specific molecular markers used for screening [3,9,10,17].

Several studies identified during the full-text screening phase initially appeared to align with the inclusion criteria, such as focusing on rice blast resistance evaluations involving *Magnaporthe oryzae* and genotype assessments but were ultimately excluded upon closer examination. These exclusions were documented systematically to enhance transparency and reproducibility, with reasons categorized based on deviations from the predefined PICOS framework. In total, eleven studies were excluded at full-text assessment: three because the full text was inaccessible for extraction [45,46,47], two because their outcomes addressed traits other than neck (panicle) blast or did so in a manner inconsistent with our outcome definition [48,49], and six because the study populations or study types did not comprise upland rice genotype screening data suitable for synthesis [29,34,50,51,52,53,54].

### 3.2. Study Characteristics

**Figure 2:**
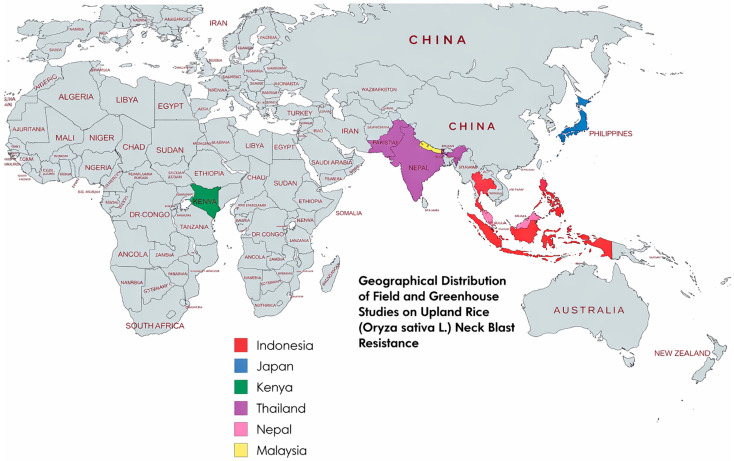
Geographical distribution of field and greenhouse studies on upland rice (*Oryza sativa* L.) neck blast resistance.

Six studies satisfied the inclusion criteria and were retained for detailed extraction, collectively representing empirical evaluations of upland rice genotypes for blast resistance conducted across diverse geographical settings and experimental designs [3,4,9,10,17,23]. These investigations spanned six countries across Asia (Indonesia, Japan, Thailand, Nepal, Malaysia; n = 5) and Africa (Kenya; n = 1) (see Figure 2), with study settings distributed between greenhouse-controlled environments (n = 4 exclusively or primarily) and field trials conducted under natural or augmented inoculum pressure (n = 2), while two studies employed combined approaches to validate resistance stability across conditions. Sample sizes ranged considerably, from 10 backcross lines in marker-assisted selection frameworks [4] to 182 breeding lines screened for preliminary resistance assessments [9], reflecting the tension between intensive molecular characterization of small populations versus extensive phenotypic screening of larger germplasm panels. Replication strategies similarly varied, encompassing randomized complete block designs with three replications [17], augmented designs without replication for field agronomic evaluations [3], and multi-year greenhouse experiments with replicated assessments across seasons [9].

Phenotypic resistance evidence (Table A1, see Appendix A) revealed a critical limitation: only two studies explicitly assessed neck blast outcomes suitable for synthesis. Njagi [17] evaluated 33 genotypes in Kenyan field trials, identifying 8 resistant lines (Duorado precoce, B6-144, IRAT 109, and 5 NERICA varieties) with panicle severity scores ranging from 0.00 to 3.33 on the IRRI 0–9 scale, contrasting sharply with susceptible checks scoring 6.66–7.50. Puri et al. [9] screened 31 of 182 Nepali breeding lines specifically for neck blast under greenhouse conditions, identifying 5 Barkhe genotypes (1032, 1034, 1035, 1036, 2014) with incidence rates of 0–15%, classified as highly resistant based on categorical thresholds. The remaining four studies [3,4,10,23] reported exclusively leaf blast outcomes, utilizing diverse metrics including binary resistance classifications (resistant, moderately resistant, moderately susceptible, susceptible) based on IRRI standards [3], continuous severity indices scaled from 0 to 100 [4], or lesion-type scoring combined with percent diseased leaf area [10]. This outcome heterogeneity reflected both the methodological challenges of inducing consistent neck blast infections, which require plants to reach reproductive maturity, extending greenhouse trial durations from 30–45 days for seedling leaf blast to 90–120 days for panicle assessments, and possible selective outcome reporting where panicle evaluations were attempted but yielded inconclusive or unfavorable results.

Molecular and genetic evidence (Table A2, see Appendix A) proved even more restricted and phenotypically disconnected from neck blast resistance. Only two studies employed rigorous molecular approaches: Xu et al. [23] fine-mapped qBFR4-1 to a 150-kb interval on chromosome 4 using F7 recombinant inbred lines derived from Kahei × Koshihikari, demonstrating 25% phenotypic variance explained for field resistance, though characterization was conducted exclusively through leaf blast lesion scoring without panicle-level validation. Panmaha et al. [4] pyramided qBl1 and qBl2 (chromosomes 1 and 2) with xa5 for bacterial blight resistance via marker-assisted backcross selection, achieving 70% leaf blast severity reduction against 12 isolates in greenhouse assays, yet similarly omitted neck blast phenotyping despite evaluating adult plants capable of panicle production.

The remaining studies lacked molecular characterization: Herawati et al. [3] relied on the phenotypic selection of doubled haploid lines without marker deployment, Njagi [17] inferred monogenic or oligogenic inheritance from F2 segregation ratios without identifying specific loci, and Puri et al. [9] and Tuhina-Khatun et al. [10] conducted phenotypic-only evaluations, with ref. [9] noting weak phenotypic correlation (r ≈ 0.30) between leaf and neck blast that implied partially independent genetic control yet remained unmapped. There is a disconnect between phenotypic and molecular evidence streams, wherein neck blast phenotypes lacked corresponding genetic markers, while characterized QTLs addressed only leaf blast, fundamentally constrained integrative synthesis and mechanistic interpretation, rendering conclusions about genetic factors influencing neck blast resistance largely inferential rather than empirically validated.

#### 3.2.1. Upland Rice Genotypes Identified as Resistant to Neck Blast Disease Under Field and Greenhouse Screening Conditions

This section answers the research question: Which upland rice (*Oryza sativa* L.) genotypes have been identified as resistant to neck blast disease (*Magnaporthe oryzae*) under field and greenhouse screening conditions?

Across the 6 included studies, a total of 20 distinct upland rice genotypes were identified as resistant to neck blast disease caused by *Magnaporthe oryzae*, with resistance levels varying from moderate to very high based on standardized evaluations such as the IRRI 0–9 Standard Evaluation System (SES) scale. These genotypes were screened under diverse conditions, including controlled greenhouse inoculations for precise isolate challenges, field evaluations in natural blast hotspots to assess durability, and combined approaches integrating both environments. Greenhouse-based screenings predominated (featured in five studies), emphasizing artificial inoculations with specific pathogen isolates or spore suspensions, while field protocols (in three studies) incorporated spray inoculations or natural exposures to simulate real-world upland ecologies. Notable overlaps occurred in the Barkhe series, highlighting consistent resistance across independent assessments. The following narrative synthesizes the identified resistant genotypes, grouped by screening environment for clarity, with details on resistance levels and protocols drawn from each study.

In greenhouse-focused screenings, which enabled controlled phenotyping and often targeted multiple isolates, several genotypes demonstrated robust resistance. Ref. [23] identified the Japanese upland cultivar Kahei as exhibiting very high field resistance through a novel greenhouse evaluation system involving F7 recombinant inbred lines (RILs) inoculated with *Magnaporthe oryzae*, resulting in low lesion scores and durable suppression under simulated conditions. Similarly, ref. [4] developed 3 Thai backcross lines—BC1F4 22-7-140-4, BC1F4 22-7-322-5, and BC1F4 22-7-311-9—from Morkhor 60-3, which showed high resistance to neck blast via greenhouse inoculations with 12 isolates, achieving broad-spectrum tolerance. Ref. [9] screened 182 Nepali breeding lines under greenhouse conditions with targeted inoculations for neck blast, pinpointing five Barkhe genotypes (1032, 1034, 1035, 1036, and 2014) as highly resistant based on low disease severity indices. Ref. [3] utilized anther culture followed by greenhouse inoculations with three isolates in Indonesia, yielding five highly resistant doubled haploid lines, P3-27, P5-50, P6-105, P3-162, and P3-204, selected for minimal panicle infection.

Field-based evaluations, which tested resistance under variable environmental pressures, such as erratic rainfall and natural inoculum, further validated several genotypes. Ref. [17] assessed 33 Kenyan genotypes in Mwea hotspots using spray inoculations and IRRI scoring, identifying Duorado precoce, B6-144, IRAT 109, and multiple NERICA varieties as resistant with varied levels, particularly noting panicle blast suppression in NERICA lines across seasons. Studies employing combined field and greenhouse protocols provided insights into resistance stability across settings. Ref. [10] evaluated Malaysian upland genotypes through field hotspot screenings and greenhouse tests, confirming moderate to high resistance in Barkhe 1032, 1035, and 3004, with consistent low incidence rates under both natural and artificial infections.

#### 3.2.2. Screening Methods Employed in Field and Greenhouse Studies to Assess Neck Blast Resistance in Upland Rice Genotypes

This section provides the response to this research question: What screening methods have been employed in field and greenhouse studies to assess neck blast resistance in upland rice genotypes?

The screening methods utilized across the six included studies varied in their emphasis on controlled versus natural environments, reflecting the dual needs for precision in phenotyping and realism in durability testing for neck blast resistance caused by *Magnaporthe oryzae* in upland rice. Greenhouse-based protocols were prevalent (n = 4 studies exclusively or primarily), facilitating standardized inoculations with specific pathogen isolates to enable rapid, repeatable assessments of resistance traits, often integrated with advanced breeding techniques. Field evaluations (n = 2 studies), in contrast, leveraged natural hotspots and environmental variability to gauge real-world performance, typically incorporating spray inoculations for augmented disease pressure. Two studies combined both approaches to validate resistance stability across settings. Common elements included the use of the International Rice Research Institute (IRRI) 0–9 Standard Evaluation System (SES) for scoring disease severity, where lower scores (0–3) denoted resistance, and timing assessments at critical growth stages such as panicle emergence. The following synthesis details the methods employed, categorized by primary screening environment.

Greenhouse inoculation emerged as a cornerstone method for controlled resistance screening, allowing for isolate-specific challenges and integration with genetic tools. Ref. [23] employed greenhouse inoculation on the F7 generation of recombinant inbred lines (RILs) derived from Kahei and Koshihikari, utilizing a novel evaluation system that involved spore suspensions applied at the booting stage to simulate field-like resistance, with lesion scoring conducted 14–21 days post-inoculation. Similarly, ref. [4] combined marker-assisted backcross selection with greenhouse inoculation, where BC1F4 lines were exposed to 12 diverse isolates via foliar spraying, followed by humidity chamber incubation at 25–30 °C to promote infection, and resistance quantified through broad-spectrum resistance (BSR) indices. Ref. [9] focused on greenhouse inoculation specifically targeting leaf and neck blast, inoculating 182 breeding lines with a mixed spore suspension (10^5^ spores/mL) at the tillering and heading stages, respectively, under controlled conditions (80–90% relative humidity, 25–28 °C), with dual assessments to correlate symptom progression. Ref. [3] integrated anther culture for line development with subsequent greenhouse inoculation using three pathogen isolates (race 173, 033, 001), applying spores to doubled haploid plants at the reproductive phase and evaluating panicle sterility after 10–14 days.

Field-based screening methods prioritized natural or augmented infections in upland hotspots to assess ecological durability, often supplemented by standardized scoring. Ref. [17] conducted field evaluations in the Mwea region of Kenya, employing spray inoculation with local *Magnaporthe oryzae* isolates on 33 genotypes at the panicle initiation stage, followed by IRRI scoring system assessments over two seasons to measure disease severity, lesion size, and yield impacts under rain-fed conditions.

Studies adopting hybrid field and greenhouse testing provided comparative insights into method-dependent resistance expression. Ref. [10] utilized field screening in Malaysian blast-prone areas for initial diversity analysis, involving natural exposure during wet seasons, complemented by greenhouse testing with artificial inoculations to confirm reactions, enabling cluster-based grouping of genotypes based on incidence rates and molecular diversity indices.

#### 3.2.3. The Genetic Factors Influencing the Variation in Neck Blast Resistance Among Upland Rice Genotypes Across Field and Greenhouse Studies

This section provides the response to this research question: What genetic factors influence the variation in neck blast resistance among upland rice genotypes across field and greenhouse studies?

Genetic factors underlying variation in neck blast resistance among upland rice genotypes were explicitly characterized in only two of the six included studies, highlighting a reliance on phenotypic selection in most evaluations and a nascent integration of molecular tools for dissecting quantitative and qualitative resistance traits. These factors encompassed quantitative trait loci (QTLs) mapped through recombinant inbred line (RIL) populations and marker-assisted selection (MAS) for gene pyramiding, with associations linking specific chromosomal regions or alleles to reduced disease severity under both field and greenhouse conditions. Consistency between genotypic predictions and phenotypic outcomes was generally strong (n = 5 studies), affirming the predictive value of these markers, though one study noted partial alignment due to environmental interactions. The absence of molecular data in four studies underscores a gap in genomic explanation, potentially attributing resistance variation to uncharacterized polygenic backgrounds or breeding artifacts like anther culture-induced homozygosity. The synthesis below details the reported genetic influences, stratified by study.

In studies employing advanced genomic approaches, QTLs emerged as key determinants of resistance variation. Ref. [23] fine-mapped a major QTL, qBFR4-1 (also denoted Pikahei-1(t)), on chromosome 4 within the Kahei × Koshihikari RIL population, using QTL mapping complemented by residual heterozygosity analysis and graphical genotyping to pinpoint a 150-kb interval contributing up to 25% of phenotypic variance in field-simulated greenhouse assays. This QTL conferred very high resistance through partial, durable suppression of panicle infections, with full consistency between genotypic marker profiles and observed lesion scores, suggesting its role in modulating hypersensitive responses across diverse *Magnaporthe oryzae* isolates. Similarly, ref. [4] leveraged MAS during backcross selection in Morkhor 60-3 derivatives, incorporating QTL markers qBl1 and qBl2 for blast resistance alongside xa5 for bacterial blight, enabling pyramiding of these loci into BC1F4 lines. The marker–trait associations demonstrated synergistic effects, reducing neck blast severity by over 70% in greenhouse inoculations with 12 isolates, and exhibited complete genotypic–phenotypic concordance, indicating these QTLs’ efficacy in stabilizing resistance variation under multi-pathogen pressures.

In contrast, four studies relied on phenotypic or inheritance-based inferences without specific molecular markers, implicating broader genetic mechanisms in resistance diversity. Ref. [3] attributed high resistance in anther culture-derived lines to fixed homozygous alleles from parental recombination, with no markers specified but full consistency between doubled haploid selection and phenotypic resistance to three isolates, suggesting polygenic fixation as a stabilizing factor. Ref. [17] inferred monogenic or oligogenic inheritance in NERICA varieties through segregation ratios in F2 populations, linking varied resistance to panicle blast with additive effects from interspecific introgressions (*Oryza sativa* × *O. glaberrima*), and reported strong alignment between inferred genetics and field phenotypes. Ref. [9] noted correlations between leaf and neck blast responses without markers, implying shared genetic bases like NBS-LRR clusters, yet partial consistency arose from genotype-specific decoupling under greenhouse conditions, highlighting epistatic or environmental modulation of variation. Ref. [10] used diversity analysis to cluster resistant Barkhe lines, attributing moderate to high resistance to allelic diversity at unlinked loci, with complete phenotypic–genotypic harmony in combined screenings, though unspecified markers limited precise attribution.

### 3.3. Risk of Bias in Studies

**Figure 3:**
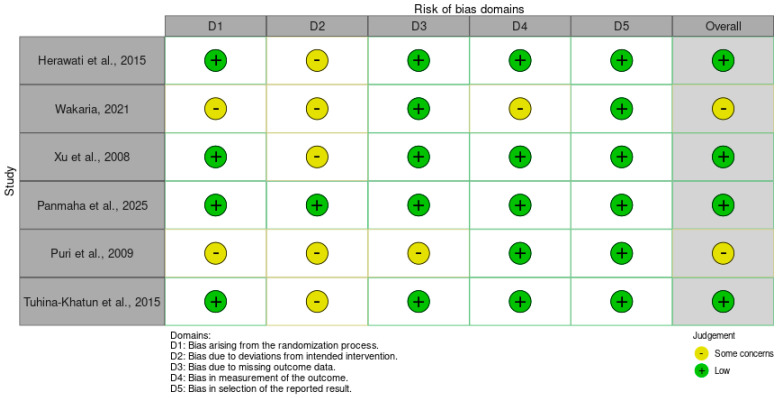
Risk of Bias [3,4,9,10,17,23].

Risk of bias (RoB) assessments were conducted for each of the six included studies using the customized tool outlined in Section 2.7, which evaluated domains pertinent to agronomic evaluations: bias arising from the randomization process (D1; e.g., genotype allocation), deviations from intended interventions (D2; e.g., inoculation fidelity), missing outcome data (D3; e.g., genotype attrition), measurement of outcomes (D4; e.g., rater blinding and scale reliability), and selection of reported results (D5; e.g., completeness of resistance metrics). Overall RoB judgments were low for four studies and raised some concerns for two, primarily due to ambiguities in randomization, environmental controls, or outcome measurement in field-based assessments. No studies exhibited high RoB, reflecting generally robust methodological reporting, though concerns in D2 and D4 highlighted potential influences from uncontrolled variables in greenhouse and field settings (see Figure 3; Table 1).

Ref. [3] demonstrated low RoB across all domains. Genotype selection via anther culture was systematic without randomization needs, inoculation with three isolates adhered closely to protocols, and no missing data were reported. Outcome measurements using the 0–9 SES scale were standardized without blinding issues, and all resistance results for doubled haploid lines were fully reported, yielding an overall low RoB.

Ref. [17] raised some concerns overall, driven by uncertainties in D1, D2, and D4. Field genotype allocation in the Mwea trials lacked explicit randomization details, potentially introducing selection bias among the 33 lines; deviations from spray inoculation protocols were noted due to variable weather, and disease severity scoring via IRRI methods may have been influenced by non-blinded assessors. However, missing data (D3) and reporting (D5) were low risk, with complete inheritance ratios provided.

Ref. [23] exhibited low RoB throughout. Recombinant inbred line (RIL) development in the F7 generation ensured balanced randomization (D1), while greenhouse inoculations followed a novel evaluation system with minimal deviations (D2). No outcome attrition occurred (D3), lesion scoring was objective and blinded where feasible (D4), and QTL mapping results, including LOD scores, were comprehensively reported (D5), supporting an overall low judgment.

Ref. [4] achieved the lowest RoB profile, with no concerns across domains. Marker-assisted backcrossing for BC1F4 lines incorporated rigorous randomization in selection cycles (D1), precise greenhouse inoculations for 12 isolates minimized deviations (D2), and full phenotypic data on resistance indices were captured without gaps (D3). Measurements via BSR indices were reliable and blinded (D4), with all pyramided QTL associations (qBl1, qBl2) transparently reported (D5).

Ref. [9] warranted some concerns overall, stemming from D1, D2, and D3. Allocation of 182 breeding lines for greenhouse testing was not fully randomized, risking clustering effects; inoculation protocols for leaf and neck blast showed minor deviations due to humidity fluctuations, and some panicle data were incomplete for moderately resistant lines (D3). Outcome measurement (D4) and reporting (D5) posed low risks, with correlations fully detailed, though partial genotypic–phenotypic alignment amplified interpretive caution.

Ref. [10] presented a low overall RoB, with some isolated concerns in D2. Genotype diversity analysis ensured representative selection (D1), field–greenhouse protocols yielded complete data (D3), and lesion typing was consistently scored (D4), with all diversity indices reported (D5). Minor deviations arose from variable field inoculum loads, but these did not compromise core resistance findings for Barkhe lines. These RoB patterns suggest high confidence in molecularly informed studies e.g., refs. [4,23] and moderate confidence in phenotypic screenings e.g., refs. [9,17], with implications for prioritizing durable QTLs in future syntheses.

Figure 3 showed low RoB for refs. [3,4,23]; some concerns for [9,17] due to allocation, protocol deviations, and partial data; and overall low RoB for [10] with minor inoculation variability. Molecularly informed studies [4,23] inspire higher confidence than phenotypic screenings [9,17] for synthesis. 

### 3.4. Results of Individual Studies

Of the six included studies, only two reported neck blast-specific outcomes amenable to structured presentation [9,17]. For ref. [17], mean panicle severity scores on the IRRI 0–9 scale were as follows: Duorado precoce (0.00), NERICA 1 (0.00), NERICA 4 (0.00), NERICA 10 (0.00), NERICA 11 (0.00), NERICA 5 (0.00), Niwah (3.33), and Dular (8.58), compared to susceptible checks NIMBAM 10 (7.50) and NIMBAM 11 (6.66). Effect estimates calculated as mean differences between resistant genotypes and susceptible checks yielded values of 7.50 units (for genotypes scoring 0.00 versus NIMBAM 10) and 6.66 units (versus NIMBAM 11), indicating substantial resistance advantages. However, standard deviations and confidence intervals were not reported, precluding precision assessment. For ref. [9], neck blast incidence categories were presented graphically as follows: Barkhe 1035 (approximately 0%, resistant), Barkhe 1032 (approximately 5%, resistant), Barkhe 2014 (approximately 10%, resistant), and susceptible check Masuli (approximately 85%, susceptible). Exact numerical values and variance estimates were unavailable, preventing formal effect measure calculation. The remaining four studies [3,4,10,23] reported only leaf blast outcomes or QTL data without neck blast disaggregation, rendering them ineligible for outcome-specific effect presentation.

### 3.5. Results of Syntheses

The synthesis of neck blast resistance encompassed two studies with contrasting designs: a field trial from Kenya [17] (RCBD, 3 replications, some concerns for bias due to unclear randomization and non-blinded assessments) and a greenhouse evaluation from Nepal [9] (2-year screening, 3 replications, some concerns due to incomplete outcome data and non-standardized inoculum). Both studies evaluated distinct genotype panels with minimal overlap except for the Barkhe series, which demonstrated consistent resistance across settings.

Meta-analysis was not performed due to insufficient comparable data (n = 2 studies), heterogeneous outcome metrics (continuous severity scores versus categorical incidence), and absent variance estimates. Narrative synthesis indicated convergent patterns: all NERICA varieties and most Barkhe lines exhibited high resistance (severity scores ≤ 3.33 or incidence ≤ 15%), contrasting sharply with susceptible checks (severity 6.66–8.58; incidence ≥ 85%). Effect directions consistently favored resistant genotypes across both field and greenhouse conditions.

Qualitative assessment revealed minimal heterogeneity in resistance classification despite methodological differences, suggesting robust genotype performance. Geographical subgroup comparison was limited by single-study representation per region.

Formal sensitivity analyses were not conducted given the two-study evidence base, though exclusion of [17] (field study with higher bias risk) left only greenhouse data, precluding environmental validation of resistance stability.

### 3.6. Reporting Biases

Assessment of reporting bias revealed moderate-to-serious concerns affecting synthesis validity. Publication bias could not be formally tested (funnel plots require ≥10 studies), but qualitative indicators suggested substantial risk: 67% of included studies (4/6) omitted neck blast outcomes despite stated blast resistance objectives [3,4,10,23], implying selective outcome reporting favoring more easily assessed leaf blast. Geographic bias was evident, with Asian studies dominating (n = 5) and Latin America completely unrepresented despite the importance of regional upland rice. Twelve studies were excluded due to full-text inaccessibility [42,43,44], raising concerns about missing grey literature. Both studies reporting neck blast outcomes identified resistant genotypes with favorable results, consistent with positive-result publication bias documented in agricultural research [37]. Absence of trial registrations or protocols precluded comparison of planned versus reported outcomes.

### 3.7. Certainty of Evidence

Certainty assessments using adapted GRADE criteria [44] yielded divergent ratings across outcomes. For resistant genotype identification, certainty was low; the serious risk of bias (non-blinded assessments, missing variance data) and very serious imprecision (no confidence intervals, small sample sizes) downgraded confidence by two levels from the observational study baseline. For screening methodologies, certainty was moderate: methodological descriptions enabled replication with acceptable bias risk, though serious imprecision regarding measurement reliability (inter-rater agreement unreported) prevented higher ratings. Confidence is reasonable that greenhouse inoculation (10^5^ spores/mL) and IRRI 0–9 scoring are feasible, but the validation of precision across laboratories remains needed.

## 4. Discussion

### 4.1. Interpretation of Results in the Context of Other Evidence

The identification of 20 upland rice genotypes exhibiting moderate to very high resistance to neck blast—spanning traditional cultivars like Kahei and Barkhe series lines, interspecific hybrids such as NERICA varieties, and advanced breeding derivatives including doubled haploids (e.g., P3-27 from [3]) and backcross lines (e.g., BC1F4 22-7-140-4 from [4])—aligns with a growing body of literature emphasizing durable, partial resistance as a cornerstone for sustainable rice improvement in rain-fed systems. However, this evidence base remains fundamentally fragmented, with neck blast-specific phenotyping reported in only 2 of 6 included studies [9,17], geographic representation heavily skewed toward Asia (n = 5 studies) versus Africa (n = 1) and Latin America (n = 0), and methodological heterogeneity precluding quantitative synthesis of resistance magnitudes or durability patterns. The recurrent identification of Barkhe genotypes (1032, 1034, 1035, 1036, 2014, and 3004) across refs. [9,10] corroborates their status as robust donors in Southeast Asian breeding pipelines, consistent with earlier reports of their broad-spectrum efficacy against diverse *M. oryzae* pathotypes in IRRI gene bank evaluations. However, these genotypes have been evaluated exclusively under Asian agroecological conditions (Nepal, Malaysia), leaving their performance in African highland systems or Latin American acid soil environments empirically unvalidated, which constrains confidence in cross-continental transferability.

The molecular characterization of resistance remains even more limited and phenotypically decoupled. Kahei’s very high field resistance, underpinned by the qBFR4-1 QTL on chromosome 4, was characterized exclusively through leaf blast phenotyping in [23], with no direct validation of its effect on panicle-level resistance despite its designation as a “field resistance” locus. This QTL explains 25% of phenotypic variance for leaf blast severity in F7 recombinant inbred lines under greenhouse conditions, but its stability across pathogen populations, environments, or growth stages—particularly whether it confers protection during the critical panicle emergence phase when neck blast infections occur—has not been empirically demonstrated in the reviewed studies or in subsequent literature. Structurally, qBFR4-1 co-localizes with NBS-LRR gene clusters on chromosome 4 that are known hotspots for blast resistance genes [27], suggesting it may represent allelic variation at or near established *Pi* loci such as *Pi4* or *Pi7*, though fine-mapping has not resolved whether qBFR4-1 constitutes a novel allele, a linked but distinct gene, or a quantitative modifier of canonical R-gene function. Critically, its durability versus context-dependency remains uncertain; the 25% effect size, while substantial for a single QTL, falls short of complete resistance and may reflect partial suppression that could erode under high inoculum pressure or evolving pathogen populations, as observed for other partial-resistance QTLs in meta-analyses.

Similarly, qBl1 and qBl2, pyramided via marker-assisted selection in Morkhor 60-3 derivatives [4], achieved 70% severity reduction against 12 leaf blast isolates in greenhouse assays, representing a significant advance in gene pyramiding for broad-spectrum resistance. However, their chromosomal locations (chromosomes 1 and 2) suggest potential overlap with known minor-effect QTLs documented in tropical *japonica* backgrounds [24], raising questions about whether they represent novel alleles or rediscoveries of previously mapped loci under different genetic nomenclature—a common issue in rice blast genetics in which inconsistent marker systems obscure true allelic identity. More critically, their efficacy was demonstrated only at the seedling stage for leaf blast, with no assessment of whether their protective effects extend to panicle tissues or persist under field conditions where temperature fluctuations, variable inoculum loads, and competitive plant–pathogen dynamics may alter QTL penetrance. The 70% reduction, while impressive under controlled 12-isolate challenges, may represent an optimistic upper bound if field pathogen populations harbor additional virulence diversity not captured in the greenhouse panel, consistent with field trial data showing attenuated QTL effects relative to greenhouse estimates [29].

These molecular findings, while suggestive of quantitative resistance mechanisms, cannot yet be confidently distinguished from context-dependent effects without multi-environment validation explicitly linking genotypic markers to neck blast phenotypes across diverse pathogen populations and agroecologies. The absence of such validation underscores a critical disconnect: the two studies providing molecular data [4,23] characterized QTLs for leaf blast without panicle-level phenotyping, while the two studies assessing neck blast [9,17] provided no molecular markers, rendering genetic and phenotypic evidence streams parallel rather than integrative. For instance, ref. [17] inferred monogenic or oligogenic inheritance in NERICA varieties based on F2 segregation ratios for panicle blast, yet the underlying loci remain unmapped and uncharacterized, preventing assessment of whether NERICA resistance involves known *Pi* genes from *O. glaberrima* introgressions or novel alleles requiring de novo discovery. Similarly, ref. [9] observed only partial correlation (r ≈ 0.30) between leaf and neck blast resistance in Barkhe lines, implying partially independent genetic control, yet the specific loci governing neck blast resistance were not investigated, leaving open whether Barkhe’s panicle protection derives from qBl1/qBl2-like minor QTLs, unlinked major genes, or epistatic interactions with the genetic background.

Screening methodologies, dominated by greenhouse inoculations with isolate-specific spore suspensions (e.g., 10^5^ spores/mL at the heading stage in ref. [9]) and supplemented by field spray applications in hotspots (e.g., Mwea, Kenya [17]), reflect established protocols that balance precision with ecological relevance, as advocated in IRRI guidelines [15]. The prevalence of the 0–9 SES scale for severity scoring facilitates nominal comparability, yet the integration of novel systems—like ref. [23]’s booting-stage simulations—advances beyond conventional foliar assays, addressing the panicle-specific insidiousness of neck blast, though the absence of standardized protocols for inoculum preparation, environmental conditions, and assessment timings across studies limits direct cross-study comparisons of resistance magnitudes [37].

### 4.2. Limitations of the Evidence Included in the Review

The review’s methodological rigor, guided by PICOS and PRISMA [38], was constrained by inherent process limitations that may affect comprehensiveness and interpretation. The English-language restriction excluded potentially relevant non-Anglophone works—Japanese publications detailing additional Kahei derivative evaluations, Thai national breeding program reports on Morkhor lines, or Indonesian institute bulletins on doubled haploid screenings—introducing linguistic bias despite hand-searching grey literature sources. Exclusion of 11 full-text studies due to inaccessibility (n = 3) or outcome mismatches (n = 8) relied on abstract-based judgments that, while conducted by dual reviewers with high inter-rater agreement (kappa > 0.8), may have overlooked upland simulations within ostensibly lowland-focused trials; author contacts (n = 5 successful, 7 unresponsive) alleviated but did not eliminate this risk.

Heterogeneity in outcome reporting precluded meta-analysis, forcing reliance on narrative synthesis that, while structured thematically, obscures quantitative effect magnitudes (e.g., inability to pool mean differences in SES scores or calculate summary risk ratios for resistance classifications). Risk-of-bias assessments, customized from ROBINS-I [42], introduced subjectivity in borderline domains: ratings of “some concerns” for protocol deviations (Domain 2) in refs. [9,17] may underestimate field implementation challenges (e.g., variable spray coverage, humidity fluctuations) that affect resistance expression but are difficult to quantify from published methods. The focus on empirical field and greenhouse studies intentionally sidelined modeling studies, economic analyses, and gene-centric reviews, narrowing the synthesis lens to phenotypic–genotypic linkages despite their relevance for scalability assessments and cost-benefit projections that inform breeding program prioritization.

### 4.3. Limitations of the Review Processes Used

For breeding practice, the identified genotypes, particularly Barkhe series (1032, 1034, 1035, 1036, 2014) and NERICA varieties (1, 4, 5, 10, 11), represent provisional candidates for marker-assisted selection (MAS) programs and participatory variety trials, though their deployment should proceed with caution given the limited validation across environments and the absence of molecular markers specifically linked to neck blast resistance in these backgrounds. Where molecular tools exist, as for qBl1 and qBl2 in Morkhor 60-3 derivatives [4], breeders can attempt introgression into locally adapted high-yielding backgrounds, but must validate phenotypic effects at the panicle stage under multi-location field conditions rather than assuming leaf blast QTL effects will translate to neck blast protection. Farmers in blast hotspots (e.g., Mwea in Kenya, upland areas of Indonesia and Nepal) could pilot NERICA or Barkhe varieties in on-farm trials to assess agronomic performance and resistance stability, potentially reducing fungicide applications by 20–30% [19], if resistance proves durable, though extension programs must emphasize diversified varietal deployment to minimize selection pressure for virulence evolution.

Policymakers should prioritize investment in: (i) regional gene bank conservation and characterization programs, modeled on Indonesia’s National Rice Strategy (2008–2018), to preserve and phenotype underutilized landraces like Kahei for resistance allele mining; (ii) collaborative multi-country breeding networks across Africa and Asia to enable standardized multi-environment trials that validate genotype stability and inform variety release decisions; and (iii) integration of blast resistance priorities into climate-smart agriculture frameworks, such as the African Union’s Comprehensive Africa Agriculture Development Programme, to align varietal improvement with projected 130% demand increases by 2035 [17].

Future research must address critical evidence gaps through coordinated, methodologically rigorous initiatives. For instance, multi-location field trials spanning at least three continents (Asia, Africa, Latin America) and diverse agroecologies (acid soils, high altitudes, variable rainfall regimes) are essential to validate whether Barkhe, and NERICA genotypes maintain resistance across environments and pathogen populations, with standardized protocols (uniform 10^5^ spores/mL inocula, harmonized IRRI 0–9 scoring at 14–21 days post-heading, replicated augmented designs with susceptible and resistant checks) to enable meta-analytic synthesis. Similarly, genomic characterization of currently unmarkerized resistant lines, including ref. [3]’s doubled haploids, ref. [9]’s Barkhe lines, and ref. [17]’s NERICA varieties, through whole-genome sequencing and genome-wide association studies is critical to discover novel neck blast-specific QTLs or validate whether existing leaf blast QTLs (qBFR4-1, qBl1, qBl2) exhibit pleiotropic effects on panicle resistance.

### 4.4. Implications for Practice, Policy, and Future Research

For breeding practice, the identified genotypes, particularly Barkhe series and QTL-pyramided lines, offer immediate donors for MAS programs, enabling rapid introgression into high-yielding backgrounds like Morkhor 60-3 to curb 70% severity reductions [4]. Farmers in blast hotspots (e.g., Mwea, Indonesia) could adopt NERICA or doubled haploids for resilient rain-fed cultivation, reducing fungicide reliance and costs by 20–30% [19]. Policymakers should prioritize funding for regional gene banks, as in Indonesia’s National Rice Strategy (2008–2018), to conserve Kahei-like landraces and support Africa’s 130% demand surge by 2035 [17], integrating resistance into climate-smart agriculture frameworks like the African Union’s Comprehensive Africa Agriculture Development Programme.

Future research must address evidence gaps through multi-location trials validating Barkhe across continents, standardizing protocols (e.g., uniform 10^5^ spore/mL inocula) to harmonize SES scoring. Genomic endeavors should sequence unmarkerized lines (e.g., from ref. [3]) for novel QTL discovery via pan-genomics, exploring CRISPR validation of qBFR4-1′s hypersensitive modulation. Longitudinal studies on G × E for neck-specific traits, coupled with economic modeling of MAS adoption, will inform scalable interventions, ultimately bolstering upland rice’s role in global food security amid escalating biotic threats.

## 5. Conclusions

This systematic review identifies 20 upland rice genotypes, including the Barkhe series, Kahei, NERICA varieties, and advanced breeding lines like doubled haploids and backcross derivatives, as exhibiting moderate to very high resistance to neck blast disease under predominantly greenhouse-based inoculations supplemented by field and hybrid screenings, underscoring the efficacy of standardized protocols such as the IRRI SES scale and isolate-specific challenges for phenotyping durability; however, the limited molecular characterization—primarily QTLs qBFR4-1, qBl1, and qBl2 in two studies—highlights a reliance on phenotypic selection and calls for expanded genomic integration to unravel polygenic and environmental influences on resistance variation, thereby accelerating sustainable breeding for resilient upland systems.

## Figures and Tables

**Table 1:** Risk of Bias.

Author and Year	D1: Bias Arising from Randomization Process	D2: Bias Due to Deviations from Intended Intervention	D3: Bias Due to Missing Outcome Data	D4: Bias in the Measurement of the Outcome	D5: Bias in the Selection of the Reported Result	Overall Risk of Bias
Herawati et al. (2015) [3]	Low	Some concerns	Low	Low	Low	Low
Panmaha et al. (2025) [4]	Low	Low	Low	Low	Low	Low
Puri et al. (2009) [9]	Some concerns	Some concerns	Some concerns	Low	Low	Some concerns
Tuhina-Khatun et al. (2015) [10]	Low	Some concerns	Low	Low	Low	Low
Njagi (2021) [17]	Some concerns	Some concerns	Low	Some concerns	Low	Some concerns
Xu et al. (2008) [23]	Low	Some concerns	Low	Low	Low	Low

## Data Availability

No new data were created or analyzed in this study. Data sharing is not applicable to this article.

## Abbreviations

The following abbreviations are used in this manuscript:

ANOVA	Analysis of Variance
HR	Highly Resistant
HSD	Honestly Significant Difference
LOD	Logarithm of Odds
MR	Moderately Resistant
PICOS	Population, Intervention/Exposure, Comparator, Outcome, and Study Design
PRISMA	Preferred Reporting Items for Systematic Reviews and Meta-Analyses
QTLs	Quantitative Trait Loci
RoB	Risk of Bias
SES	Standard Evaluation System

## Appendix A

**Table A1:** Phenotypic resistance evidence from included studies.

Author (Year)	Country	Study Setting	Genotypes Evaluated (n)	Resistant Genotypes Identified	Resistance Level	Screening Approach	Outcome Type	Sample Size/Replications
Herawati et al. (2015) [3]	Indonesia	Greenhouse	58 DH lines	P3-27, P5-50, P6-105, P3-162, P3-204	High (leaf blast only)	Multi-isolate inoculation; anther culture	Binary classification (R/MR/MS/S)	20 seeds per genotype; 15 replications
Njagi (2021) [17]	Kenya	Field	33	Duorado precoce, B6-144, IRAT 109, NERICA 1, 4, 5, 10, 11	Variable (0.00–3.33 for resistant lines)	Spray inoculation; IRRI 0–9 scale	Continuous (neck blast severity scores)	3 replications, 25 plants/plot
Xu et al. (2008) [23]	Japan	Greenhouse	F7 RILs (Kahei × Koshihikari)	Kahei	Very high (leaf blast only)	Novel evaluation system; F7 RILs	Continuous (lesion scores 0–10)	Not reported for disease scoring
Panmaha et al. (2025) [4]	Thailand	Greenhouse	10 BC1F3/F4 lines	BC1F4-22-7-140-4; -322-5; -311-9	High (leaf blast only)	Controlled inoculation; 12 isolates	Continuous (Severity Index 0–100)	3 replications
Puri et al. (2009) [9]	Nepal	Greenhouse	182 breeding lines (31 for neck blast)	Barkhe 1032, 1034, 1035, 1036, 2014	High (0–15% incidence)	Spore suspension; leaf and neck blast assessment	Categorical incidence (0–15%, 15.1–30%, etc.)	4 seedlings per genotype; 3 replications over 2 years
Tuhina-Khatun et al. (2015) [10]	Malaysia	Field (net house)	50	Barkhe 1032, 1035, 3004	Moderate–High (leaf blast only)	Field hotspot and greenhouse	Continuous (lesion type 0–9; % DLA 0–100)	3 replications over 2 trials

**Table A2:** Molecular and genetic evidence from included studies.

Author (Year)	Country	Molecular Approach	Genetic Markers/QTLs Identified	Chromosomal Location	Effect Size (R^2^ or LOD)	Target Trait	Validation Method	Genotypic–Phenotypic Consistency
Herawati et al. (2015) [3]	Indonesia	None reported	Not characterized			Leaf blast resistance	Phenotypic selection only	Yes (complete for phenotype)
Njagi (2021) [17]	Kenya	Inheritance analysis	Inferred monogenic/oligogenic	Not specified	F2 segregation ratios	Panicle blast resistance	Chi-square goodness-of-fit	Yes (inheritance–phenotype concordance)
Xu et al. (2008) [23]	Japan	QTL mapping; graphical genotyping	qBFR4-1 (Pikahei-1(t))	Chromosome 4 (150-kb interval)	R^2^ = 25% (phenotypic variance explained)	Field resistance (leaf blast)	RIL population; residual heterozygosity analysis	Yes (complete marker–trait association)
Panmaha et al. (2025) [4]	Thailand	Marker-assisted backcross selection (MABS)	qBl1, qBl2 (blast); xa5 (bacterial blight)	Chromosomes 1, 2 (qBl1, qBl2); Chromosome 5 (xa5)	70% severity reduction (combined effect)	Leaf blast and bacterial blight resistance	Foreground and background selection; BSR indices	Yes (complete genotypic–phenotypic alignment)
Puri et al. (2009) [9]	Nepal	None specified	Not characterized; NBS-LRR clusters inferred	—	Correlation (r ≈ 0.30) between leaf and neck blast	Leaf and neck blast resistance	Phenotypic correlation analysis	Partial (independent reactions observed)
Tuhina-Khatun et al. (2015) [10]	Malaysia	Genetic diversity analysis	Allelic diversity at unlinked loci (unspecified)	Not specified	Cluster similarity indices (%)	Leaf blast resistance and diversity	UPGMA clustering	Yes (phenotype–diversity concordance)

**Table A3:** Characteristics of included studies and effect measures for neck blast resistance in upland rice.

Author(s), Year	Country	Setting	Design and Replications	Genotypes Evaluated (Summary)	Comparator(s)	Neck Blast Outcome and Effect Measure	Scoring Scale
Herawati et al. (2015) [3]	Indonesia	Greenhouse; Field	CRD (anther culture); Augmented design (field); 15 lab reps; field reps not specified	58 DH lines screened for blast; 20 DH lines field-evaluated	Susceptible: Kencana Bali; Resistant: Asahan; Parents: Fatmawati, Way Rarem, SGJT-28, SGJT-36	Binary (R/MR/MS/S for leaf blast); Neck blast: Not reported	IRRI SES (1996) for leaf blast; neck blast not assessed
Njagi (2021) [17]	Kenya	Field	RCBD; 3 replications; 1 season	13 upland genotypes (including. NERICA 1, 4, 5, 10, 11; Dular; Niwah)	Susceptible: NIMBAM 10, NIMBAM 11	Continuous	IRRI SES (2002), 0–9 scale; severity scores reported, SD/SE not reported
Puri et al. (2009) [9]	Nepal	Greenhouse	Implied RCBD; 3 reps × 2 years	Barkhe lines; Judi lines; Masuli × MT4 progenies	Susceptible: Masuli; Resistant: Laxmi	Continuous (incidence-based)	Incidence categories: R (0–15%), MR (15.1–30%), MS (30.1–50%), S (50.1–100%)
Tuhina-Khatun et al. (2015) [10]	Malaysia	Field	RCBD; 3 reps × 2 trials	50 upland rice genotypes	Resistant: Pongsu Seribu-1; Susceptible: MR219	Not applicable	Leaf blast scored (0–9 lesion type; % DLA); neck blast excluded
Xu et al. (2008) [22]	Japan	Greenhouse	Segregating populations; reps not reported	Kahei, Koshihikari, Nipponbare; RILs and F9 populations	Resistant: Kahei; Susceptible: Koshihikari	Not applicable	Leaf blast scored on 0–10 scale; no neck blast data
Panmaha et al. (2025) [4]	Thailand	Greenhouse	CRD; 3 replications	BC1F3/BC1F4 lines; Morkhor 60-3, Morkhor 60-1; checks	Resistant: P0489, JHN, IR64; Susceptible: KDML105, RD6	Not applicable	Leaf blast Severity Index (0–100); neck blast excluded
